# Efficacy and Safety of JAK Inhibitors in Lichen Planus: A Systematic Review of the Available Clinical Evidence

**DOI:** 10.1155/mi/1711381

**Published:** 2026-07-22

**Authors:** Wiktoria Bajek, Anna Gwóźdź, Aleksandra Kozik, Aleksandra Spyra, Wiktor Kruczek, Aleksandra Frątczak, Anna Tekielak, Beata Bergler-Czop, Bartosz Miziołek

**Affiliations:** ^1^ Students Scientific Association, Department of Dermatology, Medical University of Silesia, Katowice, Poland, sum.edu.pl; ^2^ Doctoral School, Medical University of Silesia, Katowice, Poland, sum.edu.pl; ^3^ Chair and Department of Dermatology, Medical University of Silesia, Katowice, Poland, sum.edu.pl

**Keywords:** JAK inhibitor, Janus kinase inhibitor, lichen planus

## Abstract

**Background:**

Lichen planus (LP) is a chronic, immune‐mediated inflammatory disorder affecting the skin, mucous membranes, hair, and nails. Conventional therapies such as corticosteroids and systemic immunosuppressants often demonstrate limited efficacy and are associated with undesirable side effects. The underlying inflammatory process is largely dominated by Th1/IFN‐ɣ cytokines and the activation of the Janus kinase (JAK)‐STAT pathway, highlighting JAK inhibitors as a novel, targeted therapeutic approach for recalcitrant forms of LP.

**Aim:**

The aim of the study was to systematically analyze the current scientific evidence regarding the efficacy and safety of JAK inhibitors in the treatment of various LP variants.

**Material and Methods:**

A comprehensive literature search was performed in accordance with PRISMA guidelines using PubMed, Scopus, and Cochrane databases from inception up to May 2026. Eighty‐four relevant studies were enrolled into the final analysis. The risk of bias and quality of evidence were assessed using the JBI critical appraisal tools.

**Results:**

The final analysis consisted predominantly of case reports, case series, and limited controlled trials. Both systemic agents (e.g., tofacitinib, upadacitinib, and baricitinib) and topical formulations (e.g., ruxolitinib) demonstrated rapid reductions in pruritus and pain, followed by clinical resolution of inflammatory lesions. High rates of complete and partial clinical responses were observed across diverse variants. Short‐term adverse effects were generally mild and manageable, although the overall certainty of the evidence was low.

**Conclusions:**

JAK inhibitors demonstrate a promising preliminary clinical response and acceptable short‐term safety for managing refractory LP variants. While these agents present a valuable alternative to conventional therapies, the current evidence relies heavily on observational data. Large, randomized controlled trials with long‐term follow‐up are needed to establish optimal dosing, confirm safety, and evaluate sustained efficacy in clinical practice.

## 1. Introduction

Lichen planus (LP) is a multiform, immune‐mediated inflammatory dermatosis that can affect skin, hair, nails, and mucous membranes. It generally occurs between the third and sixth decades of life, with no strong predilection for sex or race, with the exception of oral LP (OLP), which mostly affects women (60%–70% of patients). The estimated incidence ranges from 0.14% to 1.27% in the general population, with an overall prevalence of ~1.5%. Skin lesions usually present acutely as pruritic, flat‐topped, polygonal, purple papules appearing on the extremities. Dermoscopy imaging often shows characteristic lacy, reticular, and white scales known as Wickham’s striae. Although cutaneous forms tend to resolve spontaneously in 6–18 months, other manifestations often have a chronic course. According to the EADV guidance, standard treatment of cutaneous forms of LP consists of topical and systemic corticosteroids, intralesional injections with triamcinolone, acitretin, and cyclosporine. Refractory cases may require narrowband UVB phototherapy, a combination of UV and acitretin, topical calcineurin inhibitors, and sulfasalazine [1].

The exact immunopathogenesis of CLP is not fully explained yet; however, there is some evidence suggesting that Th1/IFN‐ɣ dominates the inflammatory process in CLP. In order to have the opportunity to initiate target therapy, key immunological factors have to be identified. A recent study demonstrated expression of IFNG, IL21, IL4, IL12A, and TNF and confirmed dominance of IL‐21, IFN‐ɣ, and also pSTAT1 in the dermal infiltrate of CLP. Therefore, anti‐cytokine therapies such as Janus kinase (JAK) inhibitors may play a role in the treatment of CLP [2].

## 2. Objective

The aim of the study was to analyze the current scientific evidence regarding the use of JAK inhibitors in the treatment of LP.

## 3. Materials and Methods

**Figure 1:**
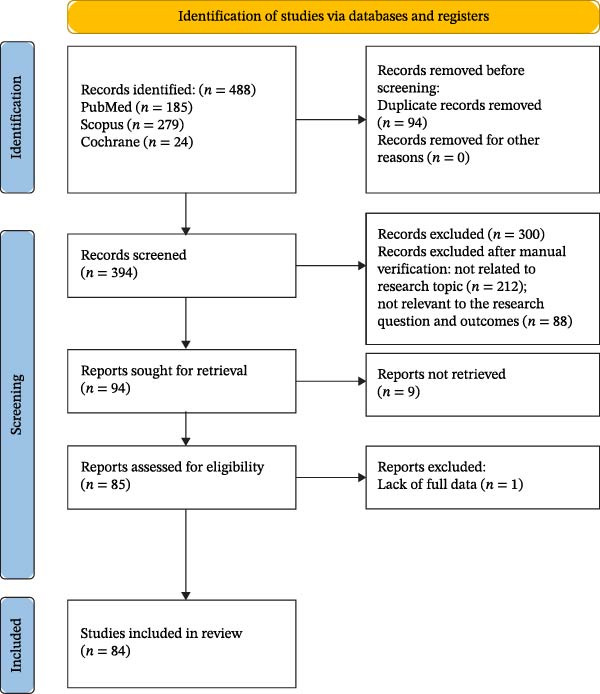
PRISMA flowchart for screening search results.

A comprehensive literature review was performed in accordance with the PRISMA guidelines (Figure 1). The search was conducted across three electronic databases from inception up to May 2026: PubMed, Scopus, and Cochrane. The search strategy was managed using the MeSH and relevant keywords. The exact search string was: ((“lichen planus” OR “lichen planopilaris”) AND (“JAK inhibitor” OR “JAK inhibitors” OR “Janus kinase inhibitor” OR “Janus kinase inhibitors” OR tofacitinib OR baricitinib OR abrocitinib OR ruxolitinib OR upadacitinib)).

Eligible studies included clinical trials, prospective observational studies, retrospective cohort analyses, case series, case reports, and research letters reporting individual patient data. To meet the inclusion criteria, studies had to evaluate either systemic or topical JAK inhibitors in patients diagnosed with any clinical subtype of LP or lichen planopilaris (LPP). Review articles, guidelines, animal or in vitro experiments, and non‐English publications were excluded.

Because research into JAK inhibitors for LP is still in an early and exploratory stage, the majority of the available evidence consisted of case reports and small case series. Reports involving only one patient were included due to the rarity of certain LP types and because dosing strategies in individual cases followed regimens established in larger studies, therefore preserving clinical relevance and allowing comparison across studies.

Each database was searched by two independent reviewers. Articles were screened manually based on their titles and abstracts. The full texts of potentially relevant articles were retrieved and independently assessed for the final decision. Any disagreements during this process were resolved through discussion and consensus, or when necessary, by consulting a third reviewer. The initial database search yielded 488 results, from which 84 were selected for the final analysis (Figure 1).

To increase the accuracy of the evidence, a formal risk‐of‐bias and quality appraisal was performed by two independent reviewers. Due to the observational and descriptive nature of the included data, the JBI critical appraisal tools were used, specifically applying the JBI checklists for case reports and case series.

## 4. Results

A total of 84 studies evaluating the clinical response and safety of JAK inhibitors in LP and LPP were included in this analysis. The literature consisted of case reports, case series, and limited cohort studies, with only a few controlled trials. The overall certainty of evidence was comparatively low, and the findings should be interpreted as preliminary clinical signals rather than confirmed efficacy. Systemic agents such as tofacitinib, baricitinib, upadacitinib, abrocitinib, and deucravacitinib were associated with rapid reductions in pruritus and pain, followed by the resolution of inflammatory lesions [3–43]. Topical formulations, including ruxolitinib, tofacitinib, and delgocitinib, also showed meaningful reductions in local inflammation and symptom severity [37, 44–51].

To standardize the heterogeneous outcomes reported across the available literature, clinical responses were categorized into complete response (CR), partial response (PR), and nonresponse (NR).

Specifically, a CR was noted when patients experienced complete or near‐complete resolution of lesions and symptoms, usually matching a greater than 75%–90% improvement in initial severity scores, lesion counts, or inflammatory activity. On the other hand, clinically meaningful but incomplete improvement was classified as PR (30%–75% reduction), while any minimal benefit under 30%, disease progression, or treatment discontinuation due to inefficacy fell into the NR group. Detailed clinical characteristics of the included studies are summarized in Table 1.

**Table 1:** Summary of the literature review on the use of the JAK inhibitors in the treatment of LP.

Study	Study design	Study group characteristics	JAK inhibitor	Dose	Outcome	Time to response	Response category	Evidence quality (JBI)
**Cutaneous lichen planus**								

Ho et al. [6]	Case report	*N* = 1 (female, 72 years)	Abrocitinib	100 mg daily	Significant pruritus reduction after 2 weeks and complete lesion resolution after 2 months	2 weeks	CR	8/8 high
Iwata et al. [7]	Case report	*N* = 1	Baricitinib	4 mg daily	Marked improvement by week 12	4 weeks	CR	8/8 high
Riekhof et al. [8]	Case report	*N* = 1	Upadacitinib	15 mg daily	Rapid itch improvement and ~ 90% clearance maintained at 1 year	24 h (pruritus) 1 month (skin lesions)	CR	8/8 high
McNamara and Tjahjono [9]	Case series	*N* = 4	Upadacitinib	15–30 mg daily	Improvement in PNRS 1–3/10 in 2–4 months, sustained results after 9 months	2 weeks	CR = 3 PR = 1	8/10 high
Mangold et al. [10]	Phase 2, randomized, double‐blind, vehicle‐controlled study	*N* = 64	Topical ruxolitinib	1.5% twice daily	Greater improvement in LiPADI, lpCAILS, and lesion count reduction versus vehicle; continued improvement through week 32	2 weeks	PR = 32	8/11 high
Barbosa et al. [11]	Case report	*N* = 1 (female, 63 year)	Abrocitinib	200 mg daily	Complete lesion clearance and pruritus resolution	1 month	CR	8/8 high
Yu et al. [5]	Case study	*N* = 1 (10‐year old boy)	Upadacitinib	15 mg	LPSI = 32 before, after 2 months LPSI = 20; mildly elevated liver enzymes	2 months	PR	8/8 high
Ball and Golant [12]	Case study	*N* = 1 (female, 52 year old)	Deucravacinb topical ruxolitinib	6 mg 1.5% cream twice daily	80% BSA with violaceous scaly plaques reduced to BSA = 50%, after 2 months BSA = 20%	5 weeks	PR	8/8 high
Hwang et al. [3]	Phase II clinical trial	*N* = 12 age: both sexes	Baricitinib	2 mg daily	Early response in 83.3%; rapid decline in the interferon signature, most prominent in the basal layer of the epidermis	2 weeks	CR = 10 NR = 2	8/10 high
Böll et al. [4]	Case series letter to editor	*N* = 7	Abrocitinib (2 patients) or upadacitinib	100 mg 15 mg	CLP activity decreased from BSA = 9.3% to 0.8%; in NRS from 7.3 to 0, in DLQI from 12 to 0	<3 weeks	CR = 7	8/10 high
Brumfiel et al. [13]	Prospective phase II study	*N* = 12	Topical ruxolitinib	Twice daily	Changes in total lesions count and changes in modified CAILS scores decreased by a mean difference of 7.6	Average 1.8 week	CR = 11 PR = 1	8/10 high

**Oral lichen planus**								

Ames et al. [14]	Case study	*N* = 1 (41‐year old female)	Upadacitinib	15 mg daily increased to 30 mg daily	Erosive OLP resolved, but reticular OLP persisted	1 month	CR	8/8 high
Noot et al. [15]	Case series	*N* = 10	Upadacitinib	15–30 mg daily	Complete clearance or marked clinical improvement in all patients	Average 45.6 days	CR = 5 PR = 5	8/10 high
Sheehan et al. [16]	Case report	*N* = 1 (female, 39 years)	Upadacitinib	15 mg daily	Complete clearance of oral ulcerations	4 weeks	CR	8/8 high
Hladky et al. [17]	Case report	*N* = 1 (female, 62 years)	Abrocitinib	200 mg daily	Complete resolution of erosive oral and vaginal lesions	3 months	CR	8/8 high
Savian et al. [18]	Case study	*N* = 1 (79‐year old female)	Baricitinib	4 mg daily	Marked improvement in ODSS from 12 to 4; complete remission after five months ODSS = 0	3 months	CR	8/8 high
Stolte et al. [19]	Case series	*N* = 3	Deucravacitib	6 mg daily	Amelioration of mucosal lesion, erosive lesions disappeared completely in 2 patients, patient three showed a strong decrease in the extent of erosive areas	12 weeks	CR = 2 PR = 1	8/10 high
Slater et al. [20]	Case study	*N* = 1 (65‐year old female)	Upadacitnib	15 mg daily	70% improvement	1 month	PR	8/8 high
Gowda et al. [21]	Case series	*N* = 5	Tofacitinib	5 mg twice daily	Significant improvement after 1 month and near‐complete resolution after 3 months with ODSS reduction in 70%	1 month	CR = 2 PR = 3	8/8 high
Mansouri et al. [23]	Case series and review of literature	*N* = 6	Tofacitinib	5 mg twice daily	Pain alleviation score VAS = 9–10 overall satisfaction VAS = 8–10; no recurrence at 6–12 months of follow‐up	Average 1.33	CR	8/10 high
Vu et al. [22]	Case study	*N* = 1 (52‐year old female)	Deucravacitinib	6 mg	Significant improvement in pain and ulcerations symptom free at 6‐month follow‐up	5 months	CR	8/8 high
Cramer et al. [24]	Retrospective multicenter study	*N* = 6	Upadacitinib, tofacitinib, baricitinib	Licensed doses	Higher excellent response rates versus conventional therapy	Months	CR = 2 PR = 1 NR = 1	8/10 high
Solimani et al. [25]	Case study	*N* = 1 (58‐year old, male)	Abrocitinib	200 mg daily	Total resolution on one side and residual symptom less Wickham striae were still visible; DLQI decreased	3 months	CR = 1	8/8 high
Moussa et al. [26]	Case study	*N* = 1 (63‐year old, female)	Baricitinib	3.4 mg twice daily	Improvement after 1 month, sustained after 4 months, near‐complete resolution of the oral irritation and discomfort	1 month	CR	8/8 high
Balestri et al. [27]	Case report	*N* = 1 (female, 45 years)	Upadacitinib	15 mg daily	Complete healing of oral lesions	7 days	CR	8/8 high
Damsky et al. [28]	Case study	*N* = 3	Tofacitinib	5 mg twice daily	Complete or near‐complete remission of the previously long‐standing and refractory ELP	NR (weeks–months)	CR = 2 PR = 1	8/10 high

**Esophageal lichen planus**								

Fikri et al. [29]	Retrospective case series	*N* = 3	Upadacitinb	15 mg daily	Clinical, histologic, and endoscopic benefits in all symptoms	NR (average evaluation 30 weeks)	CR = 3	8/10 high
Bieneck et al. [30]	Case study	*N* = 1 (56‐year old, female)	Tofacitinib	11 mg daily	After 15 months the excellent therapeutic response	12 months	CR	8/8 high
Kozlov et al. [31]	Case study	*N* = 1 (89‐year old, female)	Tofacitinib	5 mg twice daily	Consistent improvement, no AE, no recurrence	2 months	CR	8/8 high
Becker et al. [32]	Case study	*N* = 1 (68‐year old, female)	Upadacitinib	30 mg daily	Marked macroscopic and histologic improvement, with only some residual fibrostenotic disease remaining	3 months	CR	8/8 high
Landells et al. [33]	Case study	*N* = 1 (70‐year old, female)	Upadacitinib	15 mg daily	Almost complete clearance despite one erosion on tongue which increased after several months—suspicion of SCC—confirmed patient died	Months	CR	8/8 high
Kooybaran et al. [34]	Case study	*N* = 1 (77‐year old, female)	Tofacitinib	5 mg twice daily then once daily	After 24 weeks, completely free of pain and dysphagia; discontinued because of an infected hematoma	3 weeks	CR	8/8 high
Kooybaran et al. [35]	Case study	*N* = 1 (59‐year old, female)	Upadacitinib	15 mg daily	After 4 weeks the erosive free oral mucosa with no further deterioration; marked improvement in dysphagia and pain after 12 weeks; after 24 weeks oral mucosa still free of lesions	4 weeks	CR	8/8 high

**Nail lichen planus**								

Sheng et al. [36]	Case report	*N* = 1 (male, 10 year old)	Topical ruxolitinib	Twice daily	Near‐complete nail normalization	2 months	CR	8/8 high
Luo et al. [37]	Case report	*N* = 1 (female, 31 year old)	Abrocitinib	100 mg daily	Significant nail improvement	4 months	PR	8/8 high
Zhao et al. [38]	Case report	*N* = 1 (female, 33 year old)	Upadacitinib	15 mg daily	Significant nail improvement	6 months	CR	8/8 high
Iorizzo [39]	Case series	*N* = 3 (males)	Topical tofacitinib	2%	Marked improvement in NALSI with near to complete reduction in two patients; in third pterygium and longitudinal melanonychia seems to be resistant to treatment	3 months	CR = 1 PR = 2	7 /8 high
He and Yang [40]	Case study	*N* = 1 (39‐year old female)	Abrocitinib	100 mg daily (6 months) 100 mg every other day	82.35% improvement in DLQI and 79.17% NLPSI (nail lichen planus severity index)	4 months	CR	8/8 high
He et al. [41]	Case study	*N* = 1 (male)	Baricitinib	4 mg daily	Consistent improvement, with complete reduction in 6 months	2 months	CR	8/8 high
Huang and Shi [42]	Case study	*N* = 1 (41‐year old female)	Tofacitinib	5 mg twice daily	Significant improvement; no AE; still on medication with great response	6 months	PR	8/8 high
Pünchera and Laffitte [43]	Case report	*N* = 1	Baricitinib	4 mg daily	Complete nail clearance	2 months	CR	8/8 high
Iorizzo and Tosti [44]	Case report	*N* = 1 (female, 57 year old)	Tofacitinib	5 mg BID	Consistent nail improvement	6 months	PR	8/8 high

**Linear lichen planus**								

Rosenbaum et al. [45]	Case study	*N* = 1 (48‐year old female)	Upadacitinib	15 mg daily	Near‐complete reduction in 1 months and complete resolution after 6 months	1 month	CR	8/8 high

**Hypertrophic lichen planus**								

Schundler et al. [52]	Case study	*N* = 1 (male in 60 s)	Upadacitinb	15 mg daily	Complete resolution of itch and after 1 month near‐complete resolution of changes; results maintained at 1 year follow‐up	1 week	CR	8/8 high
Sharath et al. [47]	Retrospective analysis	*N* = 15	Tofacitinib	10–15 mg daily	Pruritus resolved within days; mean time of resolution of lesions—4.69 weeks; in 2 patients no improvement after 8 weeks; relapse in 5 patients; in 2 no response	8.6 day (pruritus) 4.69 week (lesion resolution)	CR + PR (*N* = 13) NR (*N* = 2)	8/10 high
Sood and Yadav [51]	Case study	*N* = 1 (48‐year old female)	Deucravacitinib	6 mg daily	Significant regression of hyperkeratotic plaques in affected areas; after 4.5 months complete resolution; results maintained at 9 month follow‐up	1.5 months	CR	8/8 high
Saha et al. [48]	Case study	*N* = 1 (23‐year old female)	Tofacitinib	5 mg twice daily	A significant improvement	2.5 month	CR	8/8 high
Youssef and Bordone [49]	Case series	*N* = 2	Tofacitinib	10 mg twice daily	P 1: after 1 month, pruritus and patches significantly improved; nonerythematous, scattered, hyperpigmented patches P 2: after 6 weeks, drastic improvement hyperpigmented patches and plaques significantly reduced in size	1–3 months	CR	8/8 high
Seiringer et al. [50]	Case study	*N* = 1 (51‐year old male)	Tofacitinib	5 mg twice daily	DLQI 23/30 before; and 4/30 after pruritus 10/10 on NRS and 0/10 after; sPGA 4/4 and 1/4 after; no AE	20 weeks	CR	8/8 high

**Inverse lichen planus**								

Ying et al. [46]	Case study	*N* = 1 (58‐year old male)	Upadacitinib	15 mg daily for 6 months; then 15 mg every other day	Significant improvement in rash, largely cleared; after 1 month follow‐up no recurrence	2 months	CR	8/8 high

**Eruptive lichen planus**								

Xue and Jiang [53]	Case study	*N* = 1 (49‐year old male)	Baricitinib	4 mg daily	After 2 weeks resolution of itching, after 20 weeks VAS changed from 4 to 0, LiPADI reduced from 26 to 14	2 weeks	CR	8/8 high

**Ulcerative lichen planus**								

Mateu‐Arrom et al. [54]	Case study	*N* = 1 (51‐year old female)	Tofacitinib	5 mg twice daily (3 weeks), then once daily (3 weeks) then 5 mg every other day	Immediate pain relief, complete capitalization; after 6 weeks purplish plaques on both soles persisted with no signs of erosion	3 weeks	CR	8/8 high
Bazargan et al. [55]	Case study	*N* = 1 (52‐year old female)	Tofacitinib	5 mg twice daily	Ulcer completely healed; no recurrence	1 month	CR	8/8 high

**Lichen planus pigmentosus**								

Sinha and Sardana [56]	Letter to editor	*N* = 1 (48‐year old female)	Tofacitinib	5 mg twice daily	Reduction in the hyperpigmentation at 12 months and near‐complete clearance of the hyperpigmentation with cessation of hairline recession at 18 months	6 months	CR	8/8 high
Li et al. [57]	Case study	*N* = 1 (35‐year old female)	Upadacitinib	15 mg daily	Marked improvement in erythematous plaques on the lower back and extremities, change in PGA from 3 to 1 and in VAS from 6 to 2; substantial reduction in pruritus	3 months	PR	8/8 high
Ira et al. [58]	Case study	*N* = 1 (24‐year old male)	Topical ruxolitinib	Topical twice daily	Overall lightening of the hyperpigmented patch on the dorsal glans penis	4 weeks	PR	8/8 high
Araghi et al. [59]	Case study	*N* = 1 (50‐year old female)	Tofacitinib	15 mg daily	Lesions improved significantly, the treatment continued until the complete resolution	1 month	CR	8/8 high
Asai et al. [60]	Case study	*N* = 1 (female)	Topical delgocitinib	0.5%	Hyperpigmentation begins to fade in 4 weeks; after 9 months almost full disappearance	4 weeks	CR	8/8 high
Bandali et al. [61]	Case study	*N* = 1 (42‐year old female)	Topical ruxolitinib	1.5% twice daily	Near‐complete repigmentation	2 months	CR	8/8 high
Cornman et al. [62]	Case study	*N* = 1 (63‐year old male)	Topical ruxolitinib	1.5% twice daily	WI‐NRS before 8/10, after 2/10	3 months	PR	8/8 high

**Lichen planopilaris**								

Ju et al. [63]	Case series	*N* = 4	Deucravacitinib	6 mg daily	LPPAI reduction of 79.6% after ≥12 weeks	≥12 weeks	PR	8/10 high
Lawrence et al. [64]	Case series	*N* = 9	Topical tofacitinib/topical ruxolitinib	Topical formulations	Partial improvement in inflammatory activity	Not reported	PR = 7 NR = 2	9/10 high
Alanazi et al. [65]	Case report	*N* = 1 (female, 29 years)	Baricitinib	4 mg daily	Complete hair regrowth maintained during treatment	2–3 months	CR	8/8 high
Lasheras‐Pérez et al. [66]	Multicenter retrospective study	*N* = 19	Upadacitini, baricitinib, abrocitinib	Standard doses	Significant reduction in LPPAI and pruritus scores	3 months	PR = 19	8/10 high
Williams et al. [67]	Research letter/retrospective cohort study	*N* = 20 (15 female, 5 male)	Ruxolitinib	1.5% cream daily	Before, LPPAI = 2.7 after LPPAI = 1.7 the average reduction in LPPAI score was 34%	Not reported	CR = 4 PR = 9 NR = 7	8/10 high
Gallo et al. [68]	Letter to the editor/case	*N* = 1 (58‐year old female)	Abrocitinib	100 mg daily	Before: LPPAI = 7, P‐NRS = 5, sleep‐NRS = 1 DLQI = 8; after: LPPAI = 0, P‐NRS = 0, sleep‐NRS = 0 DLQI = 1, the pull test (‐); complete remission of perifollicular casts and erythema	3 months	CR	8/8 high
Goodarzi et al. [69]	Observational study	*N* = 74 (62 female, 12 male)	Tofacitinib	5 mg twice daily	Before LPPAI = 4.61 ± 1.26; after LPPAI = 1.73 ± 1.68 6.75% showing no response	6–12 months	PR = 65 NR = 9	High
Mahmoudi et al. [70]	Randomized placebo‐controlled trial	*N* = 37	Tofacitinib	5 mg daily or placebo	No significant difference reduction of the LPAI; significant improvement in the anagen pull test and marked decrease in PGA score in the tofacitinib group	1 month	PR = 18	10/13 high (JBI RCT)
Janušonytė et al. [71]	Letter to the editor	*N* = 1 (73 year old)	Tofacitinib	2% cream twice daily	Resolutions of pruritus and perifollicular inflammatory lesions, effective halt of disease progression	3 months	CR	8/8 high
Gonzalez Matheus and Khosrotehrani [72]	Case report	*N* = 3 (2 female, 1 male)	Tofacitinib baricitinib	5 mg twice daily, 4 mg daily	Reduction of the size of the patches and activity of LPP, hair regrowth around the scalp vertex in one case	3 months	PR = 3	8/10 high
Fetter et al. [73]	Case report	*N* = 1 (61 years)	Topical ruxolitinib	Twice daily	Significant improvement	4 weeks	CR	8/8 high
Lasheras‐ Pérez et al. [74]	Letter to the editor/case	*N* = 5 (3 female, 2 male)	Upadacinitb	15 mg in one case, 30 mg for the rest	Before: LPPAI = 8.7 DLQI = 11 itch‐NRS = 5, 20% scalp hair loss; after: LPPAI = 1.3 DLQI = 7 itch‐NRS = 2 10% scalp hair loss	3 months	CR = 4 PR = 1	8/10 high
Li et al. [75]	Letter to the editor/case	*N* = 1 (male)	Baricitinib	4 mg daily for 3 months; after 2 mg	70% reduction of the erythema and of the hair loss; after 9 months nearly disappearance of scalp erythema	3 months	CR	8/8 high
Moussa et al. [76]	Research letter	*N* = 12 (7 with LPP, 5 with FFA)	Baricitinib	3.4 mg	Before LPPAI = 5.8; after LPPAI = 2.8 (46.5%); 2 cases with no improvement	1.5 month	CR = 3 PR = 2 NR = 7	8/10 high
Yang et al. [77]	Case series	*N* = 10	Tofacitinib	5 mg twice/three times daily	Before LPPAI = 6.22; after LPPAI = 3.08 reduction in LPPAI ranged from 30% to 94%. 2 cases with no improvement	6 months	CR = 1 PR = 7 NR = 2	8/8 high
Sallee et al. [78]	Open‐label case series	*N* = 8	Tofacitinib	5 mg twice daily; escalation to 5 mg three times daily in 2 patients	LPPAI improvement ranged from 30%–94%; relapse after withdrawal and improvement after reinitiation	Not reported	CR/PR = 8 NR = 0	6/10 moderate

**Vulvovaginal lichen planus**								

DeBiasio and Kirshen [79]	Case reports	*N* = 1 (female)	Abrocitinib	200 mg daily	95% resolution of lesions	2 months	CR	8/8 high
Benny et al. [80]	Clinical letter/case reports	*N* = 1 (female)	Tofacitinib	5 mg twice daily	Reduction of erythema and erosions	4 weeks (puritis) 16 weeks erythema and erosions	PR	8/8 high
Miao et al. [81]	Research letter	*N* = 23 (female)	Tofacitinib	5 mg twice daily	PGA 13% improved by 2 severity categories, 52.2% by one, 34.8% did not change	3.7 month	CR = 7 PR = 15 NR = 1	8/8 high
Min et al. [82]	Case reports	*N* = 1 (female)	Baricitinib, ruxolitinib	2 mg daily (4 month), then 4 mg 1.5% twice daily	Vulvovaginal lesions improved	1.5 months	CR	8/8 high
Kassels et al. [83]	Case reports	*N* = 6 (female)	Tofacitinib	5 mg twice daily	Improvement clinically and symptomatically in vulvovaginal disease	6 weeks	CR	8/10 high

**Lichen planus pemphigoides**								

Gueissaz et al. [84]	Case report	*N* = 1 (female, 56 years)	Upadacitinb	15 mg daily	Rapid pruritus resolution and near‐complete healing	1 month (itching—days)	CR	8/8 high
Balighi et al. [85]	Case reports	*N* = 1 (male)	Tofacitinib	5 mg twice daily	Significant and sustained response	2 months	CR	8/8 high
Moussa et al. [86]	Letter to editor	*N* = 1 (male)	Baricitinib	3.4 mg twice daily	Near‐complete resolution of LPP	1.5 months	CR = 3 PR = 2 NR = 7	Moderate

*Note:* LPPAI, the lichen planopilaris activity index; sleep‐NRS. Complete response (CR)—complete or near‐complete resolution of clinical lesions and/or symptoms, typically corresponding to >75%–90% improvement in disease severity scores, lesion counts, pain, pruritus, or inflammatory activity. Partial response (PR)—clinically meaningful but incomplete improvement, typically corresponding to 30%–75% improvement in disease severity scores, symptoms, or lesion burden. Nonresponse (NR)—minimal improvement (<30%), stable disease, disease progression, or treatment discontinuation due to inefficacy.

Abbreviations: CAILS, Composite Assessment of Index Lesion Severity; DLQI, Dermatology Life Quality Index; EASI, eczema area and severity index; P‐NRS, pruritus numerical rating scale; PGA, physicians’ global assessment.

### 4.1. Cutaneous LP

Both systemic and topical JAK inhibitors showed efficacy in the treatment of this variant [3–13]. Out of 105 patients treated, CR was achieved in 14.3% of cases, while PR was noted in 85.7%. Importantly, a phase II randomized controlled trial of 64 adults using topical ruxolitinib showed a significant reduction in lesion counts and higher improvement in LP activity and damage index (LiPADI) and LP composite assessment of index lesion severity (lpCAILS) scores compared to the vehicle group at week 16, with benefits continuing through week 32 [10]. Reduction and changes in skin lesions and pain were typically seen within the first few weeks of therapy in adult patients. For instance, data from a prospective phase II study using topical ruxolitinib twice daily for 8 weeks showed a reduction in the total lesion count by a median of 50 lesions, alongside a drop in the modified Composite Assessment of Index Lesion Severity score by a mean difference of 7.6 [13]. One case of a 10‐year‐old boy showed the potential of upadacitinib in the pediatric population, where the LP severity index (LPSI) decreased from 32 to 20 after 2 months of treatment; however, clinical trials on this group of patients are required for confirmation [5]. Overall, no serious adverse events were observed across the entire cutaneous LP subgroup [3–13].

### 4.2. OLP

The treatment involved deucravacitinib, tofacitinib, baricitinib, or abrocitinib [14–28]. Therapy resulted in PR or CR, marked by the healing of painful erosive lesions, and the mean time to respond was around 2 months. Specifically, patients treated with deucravacitinib experienced a complete disappearance of erosive lesions within 12 weeks, while those on tofacitinib showed a 70% reduction in the oral disease severity score (ODSS) at 3 months [21] and complete remission after 5 months [22]. Pain relief was substantial across the studies, with some case series reporting immediate pain relief scores [22, 23]. Short‐term tolerability was acceptable, with no serious adverse events reported. Only one patient treated with baricitinib experienced mild hypercholesterolemia [26], and another developed transient perioral acneiform lesions that resolved on their own [22].

### 4.3. Esophageal LP

Therapeutic responses to upadacitinib or tofacitinib were assessed [29–35]. In general, rapid pain relief was observed, and within a few weeks, disease activity decreased considerably. Patients’ mucosa was no longer erosive and remained stable during the follow‐up, with macro‐ and microscopic clearance noted within 3 months of upadacitinib use [32]. In the latest study, upadacitinib demonstrated clinical, histologic, and endoscopic benefits across all clinical symptoms [32]. Nevertheless, it is crucial to monitor patients for squamous cell carcinoma (SCC) as the skin lesions in SCC can mimic the ones that occur in esophageal LP [33].

### 4.4. Nail LP

Nail involvement showed a promising clinical response to treatment with topical tofacitinib and systemic baricitinib, tofacitinib, or abrocitinib, with clinical improvement occurring within 2–6 months. Overall, 63.6% of patients achieved CR, and 27.3% achieved PR. However, specific structural changes, such as pterygium and longitudinal melanonychia, showed lower responsiveness to JAK inhibitors, representing an NR rate of 9.1% [36–44].

### 4.5. Linear and Inverse LP

Upadacitinib, used as a therapy in both linear LP and inverse LP, led to significant improvement of symptoms within one to 2 months with no serious adverse events reported. In linear LP, an almost complete resolution of pruritus was noted within the first month, leaving only mild postinflammatory hyperpigmentation at the 6‐month follow‐up, while patients with inverse variants remained stable and relapse‐free during short‐term follow‐up [45, 46].

### 4.6. Hypertrophic LP

Oral tofacitinib and deucravacitinib led to significant improvement in skin lesions, with deucravacitinib achieving regression of hyperkeratotic plaques with complete clearance at 4.5 months [47–52]. Also, upadacitinib was reported to achieve near‐complete resolution of changes in a case report [52]. In retrospective analysis with 15 patients treated with systemic tofacitinib, pruritus resolved within days, and CR and PR were achieved in 86.7% of cases, but NR was noted in 13.3% [47]. Nonetheless, in some cases, tofacitinib seemed to be ineffective, though it is not established if it is because of the patients’ resistance to therapy with JAK inhibitors or due to not enough duration of treatment. Mild dyslipidemia and upper respiratory tract infections were noted [47].

### 4.7. Eruptive LP

This variant managed with systemic tofacitinib and baricitinib showed rapid symptom control, a fast reduction of pruritus, and overall favorable results, but data are limited to a few patients [49, 53].

### 4.8. Ulcerative LP

Treatment with oral tofacitinib resulted in immediate pain relief and no recurrence of the disease during follow‐up. However, the results are based on isolated cases available in the literature [54, 55].

### 4.9. LP Pigmentosus

Topical JAK inhibitors, including ruxolitinib and delgocitinib, led to a considerable reduction of pruritus and hyperpigmentation, with near‐complete repigmentation over time [56–62].

### 4.10. LPP

Both systemic and topical JAK inhibitors reduced clinical disease activity and halted progressive hairline recession [63–78]. The mean LPP activity index (LPPAI) score, tracked across several studies, showed significant posttreatment reductions, alongside a decrease in both NRS pain scores and the Dermatology Life Quality Index (DLQI). Furthermore, a randomized, double‐blind, placebo‐controlled trial evaluating tofacitinib over 6 months confirmed a statistically significant improvement in the anagen pull test compared to the placebo group [68]. Different multicenter respective studies assessing the use of upadacitinib, baricitinib, and abrocitinib among 19 patients showed significant reduction in LPPAI and pruritus scores [66]. Another retrospective cohort study containing 20 patients using topical ruxolitinib noted an average reduction of 34.0% in the LPPAI score, but NR was noted in 35.0% cases [67]. An observational study with 74 patients using tofacitinib also showed decreased LPPAI, with NR being observed by only 6.8% of the study population [69].

Overall, among 200 evaluated patients, a CR was achieved in 14.0% and PR in 74.5%, while 11.5% presented with NR [63‐878].

### 4.11. Vulvovaginal LP and LP Pemphigoides

In the case of vulvovaginal LP, evidence, mainly from individual case reports and small series, showed notable symptomatic relief and clinical improvement, even in patients with long‐standing, treatment‐refractory disease [79–83]. However, the therapeutic response was not universal. One case series with 23 patients evaluating tofacitinib noted that 34.8% of patients did not show any clinical improvement [81]. Similarly, cases of LP pemphigoides treated with tofacitinib or baricitinib achieved clinical resolution [84–86].

Overall, across the evaluated LP types, JAK inhibitors demonstrated a trend toward symptom relief and notable clinical responses, with mostly mild and manageable adverse effects reported in the short term. Detailed information for each study is summarized in Table 1.

## 5. Discussion

The management of refractory LP and LPP remains a significant therapeutic challenge. Although the exact immunopathogenesis of LP has not been fully described yet, T‐cell‐mediated autoimmunity and IFN‐y signaling play a key role in the chronic inflammatory cascade, providing a rationale for JAK inhibition. This systematic review, analyzing data from 84 studies, indicates that targeting the JAK‐STAT pathway offers a promising therapeutic signal across different LP subtypes. Both systemic and topical JAK inhibitors demonstrated a noticeable clinical response, characterized by marked reductions in pruritus and pain.

However, these findings must be interpreted within the context of important methodological limitations. The included evidence consists predominantly of numerous case reports, case series, and uncontrolled observational studies. A formal risk‐of‐bias assessment using the JBI checklist revealed that the majority of these descriptive reports carry a high risk of bias. Consequently, the overall certainty of evidence is very low for all synthesized outcomes, meaning that the reported clinical responses are highly likely to overestimate the true treatment effects.

Several factors caution against overinterpretation. First, LP, particularly its cutaneous forms, can follow a fluctuating or self‐limiting course, which makes it difficult to attribute clinical improvements solely to the intervention when control groups are absent. Second, publication bias is likely substantial as positive or striking therapeutic responses are preferentially reported, while failure of the treatment or adverse events rarely makes it into print. Third, the small sample sizes and short follow‐up periods in most studies limit any long‐term look at efficacy and safety. Therefore, the apparent favorable safety profile should be considered preliminary as rare or delayed adverse events cannot be excluded.

Despite these limitations, several observations require attention. In cutaneous LP, early clinical trials and case reports involving systemic agents like baricitinib, upadacitinib, and abrocitinib, as well as topical ruxolitinib, demonstrated rapid reductions in total lesion counts and body surface area (BSA), which were often accompanied by improvements in DLQI scores [3–13]. Similarly, erosive OLP treated with deucravacitinib, tofacitinib, or abrocitinib showed clinical healing and reductions in the ODSS [14–28].

In LPP and frontal fibrosing alopecia, both topical and systemic JAK inhibition decreased the LPPAI, preventing continued hairline loss [56, 63–78]. Nail LP also showed promising clinical utility with topical tofacitinib or oral baricitinib [38–46]. However, certain established structural manifestations, such as pterygium formation or advanced longitudinal melanonychia, demonstrated low responsiveness [39].

Notable symptomatic relief and clinical resolution were also documented in rarer variants, such as esophageal, hypertrophic, inverse, eruptive, ulcerative, vulvovaginal, and pigmentosus forms, as well as LP pemphigoides [29–35, 45–62, 79–86].

The rapid relief of symptoms, often occurring within days to weeks, contrasts with the typically slower response seen with conventional therapies, such as corticosteroids. Furthermore, the consistency of clinical benefit across such diverse LP subtypes suggests a class effect for JAK inhibitors. The ongoing randomized clinical trials involving ruxolitinib and tofacitinib offer the prospect of more reliable evidence in the near future [58, 59, 62].

The identification of SCC in a patient with esophageal LP treated with upadacitinib [33] highlights the importance of careful monitoring. While malignant transformation is a known complication of chronic erosive LP no matter the treatment [87, 88], future studies should systematically evaluate whether prolonged JAK inhibition alters this risk. This case underscores that any isolated lesion demonstrating therapeutic resistance to JAK inhibitors mandates an immediate biopsy to rule out malignancies.

In conclusion, JAK inhibitors represent a promising therapeutic avenue for LP, especially in treatment‐refractory cases where therapeutic options are limited. However, because of the low certainty of the available evidence, these agents should be considered an emerging treatment option rather than a standard of care. Well‐designed, controlled trials with standardized outcome measures, adequate sample sizes, and long‐term follow‐up are urgently needed to establish their true efficacy, optimal dosing, and safety profile across the spectrum of LP subtypes.

## 6. Conclusions

JAK inhibitors, both oral and topical, show a promising preliminary clinical response in the management of various LP variants, with an acceptable short‐term safety and tolerability profile reported so far. Current clinical findings suggest their therapeutic potential for LP treatment as an alternative option for patients with refractory LP who have failed conventional systemic and topical therapies. However, because the existing literature mainly consists of case reports and small series, the overall certainty of this evidence remains low. Long‐term outcomes, recurrence rates after treatment cessation, and potential publication bias must be carefully considered, as definitive data on safety and sustained remission are still lacking.

Further studies involving larger clinical trials evaluating specific JAK inhibitors for each LP subtype are needed. Comparative data of this kind would be essential to establish optimal dosing and evaluate the long‐term efficacy and safety in clinical practice.

## Author Contributions

**Wiktoria Bajek**: conceptualization, investigation, methodology, writing – original draft. **Anna Gwóźdź, Aleksandra Kozik, and Aleksandra Spyra**: investigation, writing – original draft. **Wiktor Kruczek and Aleksandra Frątczak**: project administration, resources, validation, visualization, writing – review and editing. **Anna Tekielak**: writing – review and editing. **Bartosz Miziołek and Beata Bergler-Czop**: supervision, coordination.

## Funding

This study received no external funding, but the Open Access costs were covered thanks to the support of the Medical University of Silesia in Katowice under agreement BNW‐NWN‐640‐2‐1‐349/26.

## Disclosure

Bartosz Miziołek and Beata Bergler‐Czop approved the final manuscript for submission.

## Ethics Statement

The authors have nothing to report.

## Conflicts of Interest

The authors declare no conflicts of interest.

## Data Availability

The data that support the findings of this study are available from the corresponding author upon reasonable request.
